# Combining Taipan snake venom time/Ecarin time screening with the mixing studies of conventional assays increases detection rates of lupus anticoagulants in orally anticoagulated patients

**DOI:** 10.1186/1477-9560-5-12

**Published:** 2007-09-06

**Authors:** Gary W Moore

**Affiliations:** 1 Centre for Haemostasis and Thrombosis, (The Haemophilia Reference Centre), St. Thomas' Hospital, London, UK

## Abstract

**Background:**

Oral anticoagulation compromises conventional lupus anticoagulant (LA) screening assays. Mixing studies can counteract the oral anticoagulant effect but the dilution reduces sensitivity and can generate false negative results. A firm diagnosis can be made from mixing studies when an elevated screen ratio is accompanied by a confirm ratio that generates significant correction to demonstrate phospholipid dependence, but also returns into the reference range, indicating complete normalisation of the oral anticoagulant effect. Taipan snake venom time (TSVT) with Ecarin time (ET) as a confirmatory test comprises an oral anticoagulant insensitive LA detection system and this study investigates the potential impact on detection rates when coupled with mixing studies on standard assays.

**Methods:**

Eighty patients known to have LA who were receiving oral anticoagulation were tested with TSVT/ET and 1:1 mixing studies with normal plasma by dilute Russell's viper venom time (DRVVT) and dilute activated partial thromboplastin time (DAPTT) to assess detection rates by single and multiple assays.

**Results:**

Thirty three of the 80 samples from known LA positive patients were positive in all three assays and 15 were positive in combinations of DRVVT, DAPTT or TSVT/ET. The remainder were positive in only one assay; 12 by DRVVT, 4 by DAPTT and 16 by TSVT/ET. Although all DRVVT and DAPTT positive mixing studies generated significant correction of the screen ratio by the confirm ratio, not all confirm ratios corrected back into the reference range. This was the case for 87.5% of the DRVVT results, 44.7% of the DAPTT results and 13.3% of the TSVT/ET positive mixing tests.

**Conclusion:**

Addition of TSVT/ET screening for LA in orally anticoagulated patients could increase diagnostic efficacy either by detecting antibodies diluted in the mixing tests of conventional assays or those that do not react in DRVVT or DAPTT. Additionally, TSVT/ET can affirm the presence of a LA where conventional assay mixing tests may not have fully counteracted the oral anticoagulant effect but confirmatory test correction suggests the presence of a LA.

## Background

Lupus anticoagulants (LA) comprise part of the heterogeneous spectrum of acquired autoantibodies termed antiphospholipid antibodies (APA) [1]. The occurrence and persistence of APA is associated with a wide range of clinical signs and symptoms, most commonly arterial and venous thrombosis, recurrent foetal loss and thrombocytopenia [2].

LA are identified in the laboratory by their interference with one or more phospholipid dependent coagulation assays [3]. Although both national [4] and international guidelines [5] have been published in recent years for the laboratory detection of LA, their identification remains a problem, in part due to the limitations in sensitivity and specificity of current methodologies [6,7]. Due to the heterogeneous nature of the antibodies, more than one LA sensitive test should be used, of different assay types, in order to maximise detection rates when screening for LA [5,6]. Some LA react better in certain test systems than others or may only be detected in one type of assay [5].

Oral anticoagulant therapy (OAT) can compromise LA testing on neat plasma so a commonly adopted approach is to perform screen and confirm assays on 1:1 mixtures with normal plasma [4]. This will correct the OAT effect in many cases [1] but the dilution of the antibodies reduces sensitivity and can lead to false negative results [8]. As in neat plasma, an elevated screen accompanied by significant confirmatory test correction indicates the presence of a LA, although caution is indicated where the confirmatory test does not additionally return into the reference range as this may indicate incomplete normalisation of the oral anticoagulant effect [1]. Taipan snake venom time (TSVT) screening using the Ecarin time (ET) as a confirmatory test is a sensitive assay system for detecting LA in patients on OAT [9] and the present study assesses whether combination of TSVT/ET analysis with mixing studies on dilute Russell's viper venom time (DRVVT) and dilute activated partial thromboplastin time (DAPTT) could increase detection rates.

## Methods

### Blood collection, manipulation and storage

Blood was collected into a one tenth volume of 0.105 M tri-sodium citrate and double centrifuged to obtain plasma with a platelet count of less than 10 × 10^9^/L as previously described [10]. The platelet poor plasma for LA testing was stored at -70°C for no longer than 2 months and thawed at 37°C for 5 minutes prior to analysis. Plasma for coagulation screening was analysed fresh, immediately after centrifugation.

### Coagulation screening tests

Coagulation screen comprising prothrombin time, reported as international normalised ratio (INR), activated partial thromboplastin time, thrombin time and Clauss fibrinogen were performed on a Sysmex CA 1500 (Sysmex UK Ltd, Milton Keynes, UK) using Innovin^® ^recombinant thromboplastin, Actin FS^®^, Thromboclotin^® ^and Thrombin-Reagent^® ^(Dade-Behring, Marburg, Germany). Coagulation screens were performed to assess ongoing anticoagulant therapy and help exclude heparin therapy or coagulopathies that could mask, mimic or co-exist with LA.

### Lupus anticoagulant assays

LA screening was undertaken on the Sysmex CA1500 analyser using three assays. DRVVT was performed with Gradipore LA Screen and LA Confirm reagents (BioMérieux UK Ltd, Basingstoke, UK). DAPTT was performed using PTT-LA (Diagnostica Stago, Asniéres, France) in the screen with a platelet neutralisation procedure employing Biodata Platelet Extract Reagent (Alpha Laboratories, Hampshire, UK) in the confirmatory test. TSVT/ET analysis was performed using Diagen Taipan venom (Diagnostic Reagents, Thame, UK) and *E. carinatus *venom (Diagnostic Reagents) using an automated version of the previously described method [9]. All elevated screens received the confirmatory test plus a screen and confirmatory test on 1:1 mixing studies with normal plasma [2,3]. Lyophilised Platelet Poor Plasma, (Technoclone, Dorking, UK) was used as the normal plasma throughout. It is specifically manufactured to be sufficiently platelet poor for use in LA assays by careful handling and repeated centrifugation.

### LA assay result interpretation criteria

DRVVT, DAPTT and TSVT screen and confirm results were converted to ratios by dividing the clotting time of the test by that of the normal control. Interpretation of the data for the presence of a LA was made by calculating the percentage correction of the screening test ratio by the confirmatory test ratio. Test plasmas were defined as being consistent with the presence of a LA if the screening test ratio was greater than the upper limit of normal and this was corrected by ≥ 10% [4,8-10]. In view of the potential compromising effect of OAT on DRVVT and DAPTT in neat plasma, only the results from 1:1 mixing studies were assessed for LA activity [4]. Taipan and Ecarin venoms are largely unaffected by OAT [9] so both neat plasma and mixing study results were evaluated provided that other causes of prolonged clotting times were excluded [4,5]. Reference ranges for screen and confirm assays in neat plasma and 1:1 mixing studies, calculated as ± 2 standard deviations of the mean were previously locally derived [4,8,11] from 40 normal donors [4] with normal clotting screens and no evidence of haemostatic disease.

### Patients

DRVVT and DAPTT mixing test and TSVT/ET results on 80 patients known to have LA [4,5] who were receiving OAT and were positive in at least one of those assays whilst anticoagulated were assessed to ascertain frequencies of positivity in each test, and thus, whether addition of TSVT/ET analysis to a conventional LA detection strategy could increase detection rates in the diagnostic setting. The study was a retrospective analysis of data generated for disease/treatment monitoring and all patients gave verbal consent.

## Results

The INRs on the 80 LA patients receiving OAT were within the range 1.23 – 4.77. Twenty five had INRs below the therapeutic range of 2.00 – 4.50 and two were above it. There was no correlation between degree of INR prolongation and screening test value in any of the LA assays.

A total of 56 of 80 (70%) samples were positive in DRVVT mixing tests, 47 of 80 (58.8%) in DAPTT mixing tests and 58 of 80 (72.5%) in TSVT/ET on neat plasma. The frequencies of LA positivity with single assays and combinations of assays for the 80 LA positive patients on OAT are shown in Table 1.

**Table 1:** Frequencies of lupus anticoagulant positivity with different assay combinations in 80 orally anticoagulated patients with lupus anticoagulants

	Assay combinations						
	
	DRVVT, DAPTT & TSVT	DRVVT & DAPTT	DRVVT only	DAPTT only	TSVT/ET only	TSVT/ET & DRVVT	TSVT/ET & DAPTT
Number positive for LA (%)	33 (41.3)	6 (7.5)	12 (15.0)	4 (5.0)	16 (20.0)	5 (6.2)	4 (5.0)

LA, lupus anticoagulant; DRVVT, dilute Russell's viper venom time; DAPTT, dilute activated partial thromboplastin time; TSVT, Taipan snake venom time; ET, Ecarin time

The diagnosis of the presence of a LA in mixing studies is firmer if the confirmatory test ratio not only corrects by ≥ 10% but back into the reference range, as this indicates complete correction of the OAT effect. The frequencies of correction back into the mixing test specific confirmatory test reference range or by ≥ 10% but not into the reference range are shown in Table 2.

**Table 2:** DRVVT, DAPTT and TSVT mixing study data from 80 orally anticoagulated patients with lupus anticoagulants

Assay	n	Number with confirmatory test correction back into reference range (%)	Number without confirmatory test correction back into reference range (%)
DRVVT	56	7 (12.5)	49 (87.5)
DAPTT	47	26 (55.3)	21 (44.7)
TSVT/ET *	58	26 (44.8)	4 (6.9)

* Twenty eight of 58 (48.3%) TSVT mixing studies had TSVT ratios within the mixing study specific reference range and were excluded from this analysis. DRVVT, dilute Russell's viper venom time; DAPTT, dilute activated partial thromboplastin time; TSVT, Taipan snake venom time; ET, Ecarin time

## Discussion

Therapeutic anticoagulation inevitably compromises many coagulation assays that are designed to detect a specific abnormality based on the assumption that the patient's coagulation is otherwise normal. Use of heparin neutralisers and mixing studies are available to counteract these effects but they are not without their limitations and accurate detection may not be possible [4,8,12]. Whilst it is rarely necessary to investigate for LA in heparinised patients it is relatively often required in those who are receiving OAT [4]. The most frequently used assays to detect LA in the UK are DRVVT and a variety of APTT based assays [13]. Testing on neat plasma in these assays is compromised by OAT giving rise to false-negative and -positive results in patients with and without LA [4,14,15] and mixing studies are commonly used to correct the acquired multiple factor deficiency of OAT.

Although the relatively small dilution in 1:1 mixing studies can dilute LA to undetectable levels in a significant number of cases [8], it remains a useful tool for demonstrating the antibodies in the presence of co-existing coagulopathies providing that the LA is sufficiently potent to prolong the screening test and a confirmatory test is used to demonstrate phospholipid dependence. Fifty six of 80 known LA were detected in the DRVVT mixing tests and 47 of 80 by DAPTT mixing tests, so they clearly have a role to play in detection by commonly used conventional assays. As would be expected, some antibodies were only apparent in one of either DRVVT or DAPTT, more so in DRVVT. However, incomplete normalisation of the OAT effect can be encountered in patients on high dose OAT [1,16] so diagnosis of the presence of a LA is more reliable when the mixing study confirmatory test corrects back into the reference range whilst the screen remains elevated. Significantly, a very low percentage of DRVVT positive LA (12.5%) generated a mixing study confirmatory test correction into the reference range, in contrast to the 55.3% by DAPTT and 44.8% by TSVT/ET. This is likely due to the specific analyser/reagent/normal plasma combination [14] with this patient population and would not necessarily be true for other DRVVT/analyser combinations. Incomplete normalisation of the confirmatory test can also be attributable to avid antibodies [17,18], but providing that additional coagulopathies are excluded, an elevated screen in mixing studies with ≥ 10% correction by a confirmatory test result above the reference range suggests the presence of a LA. Some such antibodies appear to possess a degree of resistance to the swamping effect of high phospholipid confirmatory reagents. This may explain why so few of the TSVT/ET mixing studies with elevated screens did not correct back into the reference range as the ET is a phospholipid independent reagent. Clear positivity in TSVT/ET screening can affirm a suggestive diagnosis in conventional mixing studies. Of note is the 48.3% of TSVT/ET positive LA that were undetectable in mixing studies compared to the 39.3% previously reported for this assay system [8], further emphasising the limitations imposed by the dilution effect.

Venoms from the Coastal Taipan (*Oxyuranus scutellatus*) and the Saw-scaled viper (*Echis carinatus*) contain prothrombin activators capable of activating the des-carboxy prothrombin produced during OAT as well as native prothrombin. The Taipan activator is phospholipid and calcium ion dependent but the Saw-scaled viper activator is not. Together they represent an alternative or adjunct to conventional assay mixing tests in detection of LA patients on OAT. As the DRVVT and DAPTT positive LA were detected using mixing studies, it is unsurprising that more than half were also detectable by TSVT/ET as they would have been potent antibodies and more likely to present in multiple assays. Nevertheless, 27.5% were not detected in TSVT/ET, a clear manifestation of antibody heterogeneity, and likewise, those LA that were positive in TSVT/ET and just one of either DRVVT and DAPTT. Of particular relevance to this study are the 20.0% that were detectable only by TSVT/ET. Irrespective of whether these LA were TSVT/ET detectable only and/or would have presented in DRVVT and/or DAPTT without the OAT effect, there are clear implications for initial diagnostic LA screening on patients receiving OAT. Extrapolating these findings in known LA to the diagnostic setting, use of TSVT/ET would increase detection rates by detecting LA that would be diluted out in DRVVT and DAPTT mixing tests, and also those that are undetectable in conventional assays, probably as a result of antibody heterogeneity and epitope specificity. This should not however engender a false sense of security as some LA that are only detectable in DRVVT or DAPTT will be negative in TSVT and diluted in mixing studies.

Additionally, spontaneous variation of APA has been reported to occur in up to 25% of cases [19] and TSVT/ET analysis could have a role in disease monitoring for patients on OAT whose antibody presentation or avidity changes over time [20].

## Conclusion

There is sufficient evidence to suggest that conventional screening for LA on neat plasma in patients receiving OAT is unreliable. Mixing studies are widely used and can detect LA in some patients but are not undertaken by all laboratories [13], resulting in the possibilities of either inaccurate diagnostic outcomes or non-performance of LA screening in this patient group. TSVT/ET screening can detect LA missed in conventional assays due to non-reactivity in those tests or dilution to undetectable levels in mixing studies. In other patients where the mixing tests are positive but the confirmatory assay does not correct back into the reference range, clearly positive TSVT/ET results affirm diagnosis. Although TSVT/ET mixing studies are unnecessary for correction of the OAT effect, they should still be used with this assay system alongside testing on neat plasma to demonstrate inhibition and enhance specificity. Addition of TSVT/ET screening to an existing repertoire of conventional assays has the potential to improve detection rates in a group of patients where LA identification is difficult.

## Competing interests

The author(s) declare that they have no competing interests.
